# Anatomical Markers for Identification and Standardization of *Crataegus mexicana*, Commercially Marketed as “Raíz de Tejocote”

**DOI:** 10.3390/plants14233607

**Published:** 2025-11-26

**Authors:** Sebastian J. Adams, Laura Estupiñán-Pérez, Gloria Melisa González-Anduaga, Andrés Navarrete, Ikhlas A. Khan

**Affiliations:** 1 National Center for Natural Products Research, School of Pharmacy, The University of Mississippi, Oxford, MS 38677, USA; 2 Department of BioMolecular Sciences, School of Pharmacy, The University of Mississippi, Oxford, MS 38677, USA; 3 Facultad de Química, Departamento de Farmacia, Universidad Nacional Autónoma de México, Ciudad Universitaria, Coyoacán, Ciudad de México 04510, Mexico; lauriest.0901@gmail.com (L.E.-P.); mel_doux@hotmail.com (G.M.G.-A.)

**Keywords:** *Crataegus*, dietary supplements, herbal products, Mexican hawthorn, microscopy, authentication, quality control, Raíz de Tejocote, weight loss

## Abstract

Background: “Tejocote, manzanita, tejocotera”, and Mexican hawthorn are the popular common and commercial names of *Crataegus mexicana* Moc. & Sessé ex DC. This medicinal and edible plant species is widely used for weight loss and for treatment of cardiovascular, inflammatory, neurological, and respiratory infections. Several commercial products are marketed as “Raíz de Tejocote” for weight loss; however, these are frequently adulterated with other plants, other *Crataegus* species, or other parts of genuine *C. mexicana*. In this sense, this work aims to provide the anatomical features of the leaf and stem, and especially to authenticate the root of *C. mexicana*. Methods: The study utilized light microscopy, fluorescence microscopy, scanning electron microscopy, and energy-dispersive X-ray spectroscopy to identify the key characteristics that differentiate the market sample sold under the name Raíz de Tejocote. Results: Anatomical features revealed that the sample sold as Raiz de Tejocote is not a root but a stem. The absence of key diagnostic features such as cork, cortex, cambial layers, and sclereids in the cortex, and the presence of pith, uniseriate rays, radial vessel patterns, and clustered pits, strongly suggests that the market sample is adulterated, most likely derived from a stem of a *Crataegus* species, but not the *C. mexicana*. Conclusions: The anatomical comparison indicates that the market sample does not match the root or stem characters of *C. mexicana*. This comparative anatomical profiling can serve as a reliable authentication parameter, especially if the sample is taken for quality check as a whole, cut and sifted, or coarse powder form, based on the wood characteristics, xylem vessel and fiber characteristics provided.

## 1. Introduction

The genus *Crataegus* L. comprises over 200 species, distributed in the temperate regions of the Northern Hemisphere [1]. Taxonomically, this genus is considered highly complex in its phylogenetic classification due to extensive morphological variation, hybridization, and subspecies. These factors continue to challenge taxonomists, making this genus an attractive subject for ongoing research aimed at establishing a more solid and reliable basis for its classification. This genus comprises 13 species distributed across regions of Mexico, among which *Crataegus mexicana* Moc. & Sessé ex DC., commonly known as Mexican hawthorn or tejocote [2] belongs to the family Rosaceae Juss., subfam. Amygdaloideae Arn., tribe Maleae Small. This species is distributed in Guatemala and Mexico (Guanajuato, Hidalgo, Jalisco, México, Michoacán, Oaxaca, Puebla, Querétaro, San Luis Potosí, and Veracruz), and it is widely cultivated in Costa Rica, Ecuador, and El Salvador [3,4]. It is a deciduous fruit-bearing tree of considerable ethnobotanical and medicinal significance in Mesoamerican cultures. The main useful parts of this plant are the fruits, leaves, and roots, which are mainly used in traditions of the Nahua, a large group of Indigenous peoples of Mexico and El Salvador, and they were used for treating gastrointestinal ailments [5,6,7,8]. Tejocote (derived from the Nahuatl language Texocotl, from “tetl” meaning “hard” and “xocotl” meaning sour; “sour fruit” or “hard fruit”) is also culturally significant during Mexican festivities, notably in “Día de los Muertos” and Christmas “posadas,” where the fruits are incorporated into dishes, syrups, hot beverages, and used to fill piñatas [8,9].

Phytochemical studies on this species are limited. An analysis by HPLC of the fruit (peel and pulp) revealed the presence of epicatechin, quercetin 3-*D*-galactoside, ascorbic acid, procyanidin B2, and chlorogenic acid, while vitexin and catechin were identified in the seeds [10]. Recent research has reported three different dibenzofurans in the root: 2, 3, 4, 7-tetramethoxydibenzofuran-5-ol; 2, 3, 4-trimethoxydibenzofuran-5, 6-diol, and 1,2,3,6-tetramethoxy dibenzofuran [11,12]. There are reports on the close species of *Crataegus*, that show that chemicals such as chlorogenic acid, (+)-catechin, (−)-catechin, quercetin, kaempferol, and apigenin glycosides have been identified in the ethanolic extracts from the fruits of *C. nelsonii* Eggl. *C. stipulosa* (Kunth) Steud. (syn. of *C. mexicana*), and *C. pubescens* Steud [9,13].

Despite its valued role in traditional medicine and nutrition, *C. mexicana* faces issues of adulteration and misidentification of dried roots or fruit-based herbal products in commercial markets. Other common *Crataegus* species, such as *C. laevigata* (Poir.) DC. (Midland hawthorn), *C. monogyna* Jacq. (European hawthorn), *C. pubescens* (syn. of *C. gracilior* J. B. Phipps), and other species like *C. arnoldiana*, and *C. douglasii* were used in other parts of the world as well [14]. The species sold as Raíz de Tejocote, is mainly misidentified or adulterated with non-*Crataegus* sp. plants and is used in herb-based products, potentially compromising both efficacy and safety. Recently, several cases have been reported concerning the safety of *C. mexicana* products sold for weight loss, which were found to be adulterated with the nuts of yellow oleander, botanically called *Cascabela thevetia* (L.) Lippold. (Family Apocynaceae) [15,16]. Products containing *Thevetia peruviana* (Pers.) K. Schum. (syn. of *Cascabela thevetia*) have been banned in many countries, like the United States of America (USA), South Korea, and the United Arab Emirates (UAE), and warnings have been issued by national authorities, such as U.S. Food and Drug Administration (FDA); the National Health Surveillance Agency (ANVISA), Brazil; and the Federal Committee for Protection from Sanitary Risks (COFEPRIS) of the Mexican Ministry of Health, regarding its use in traditional herbal medicine and dietary supplement products due to due to the presence of toxic cardiac glycosides such as thevetin A, thevetin B, and neriifolin [17,18]. This emphasizes the importance of botanical authentication through macro- and microscopic validation as the essential steps to verify that the correct species and plant parts are used before chemical profiling is conducted. It is also crucial to follow the guidelines set by the U.S. Food and Drug Administration (FDA) under 21 CFR Part 111, “Current Good Manufacturing Practice in Manufacturing, Packaging, Labeling, or Holding Operations for Dietary Supplements,” to maintain the integrity of herbal preparations and protect public health. However, for *Crataegus* species, especially those sold commercially as tejocote, there is a significant lack of clear key anatomical features that can reliably distinguish authentic market samples from adulterated ones. Existing sources, such as the Inside Wood database (https://insidewood.lib.ncsu.edu/ accessed on 7 August 2025), provide anatomical descriptions of wood; however, detailed comparative studies are limited and inadequate for solid authentication using wood, root parts, and leaves. Therefore, this study aims to develop a thorough anatomical profile for *Crataegus*, identifying diagnostic characteristics to differentiate genuine samples from whole or chopped forms of substitutes or adulterants in the commercial herbal market.

## 2. Results and Discussion

### 2.1. External Morphology of C. mexicana Plant Parts and Marketed Tejocote Root

**Figure 1:**
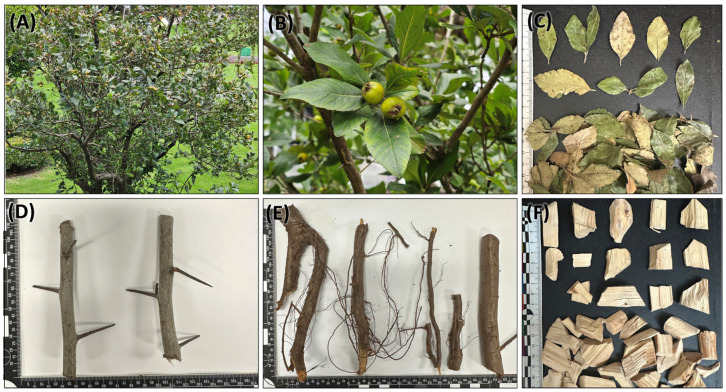
External morphology of *C. mexicana*. (**A**) Plant habit, (**B**) closer view of leaves and fruits, (**C**) dried leaves, (**D**) stem, (**E**) root, and (**F**) market sample sold as Raíz de Tejocote.

This species is a large shrub or small tree, typically attaining heights of up to 10 m, with stout and robust branchlets (Figure 1A,B). The leaves are lanceolate to elliptic, occasionally narrowly obovate in outline. The abaxial surface is densely tomentose, while the adaxial surface is glabrous to sparsely pubescent. Leaf margins are serrate to serrulate (Figure 1C). The dried leaves are stiff and brittle when crushed, yet they generally remain intact with the margins well preserved in the raw sample. The dried stems are often thorny, and pale green to brown in color (Figure 1D). The dried root part is dark brown in color, and the inner wood is brownish red in color (Figure 1E). The market samples (dried) consist mostly of cut and sifted stem pieces with the bark removed (Figure 1F). These pieces are lightweight and pale whitish in color, indicating that the sample being sold is not a root part and is also not, based on its color, a genuine stem of *C. mexicana*.

### 2.2. Microscopic Description of C. mexicana and Market Sample Characteristics

#### 2.2.1. Leaf Anatomy of *C. mexicana*

**Figure 2:**
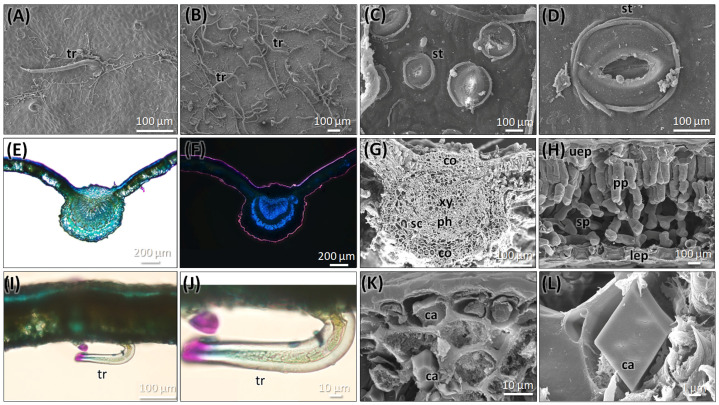
Micromorphology of the leaf of *C. mexicana*. (**A**) Upper surface, (**B**) lower surface, (**C**,**D**) the presence of stomata on the lower surface of the leaf (**E**,**F**). Light microscopic observation of the transverse section of the leaf, under bright wide-field illumination (**E**), stained with TBO; (**F**) is viewed under epifluorescence. (**G**,**H**) SEM image of the cross-section of a leaf showing the leaf’s anatomical structure. (**I**,**J**) Light microscopic observation of the transverse section of the leaf showing the trichome. (**K**,**L**) SEM image of calcium oxalate crystals presents in the leaf. ca: calcium crystals, co: collenchyma cells, lep: lower epidermal layer, ph: phloem, pp: palisade parenchyma, sc: sclerenchyma, sp: spongy parenchyma. st: stomata, tr: trichome, uep: upper epidermal layer, and xy: xylem. Scale bars: (**E**,**F**) 200 µm, (**A**,**B**,**I**) 100 µm, (**C**,**D**,**G**,**H**,**J**,**K**) 10 µm, and (**L**) 1 µm.

The leaf of this species is simple, alternately arranged with an ovate to obovate shape with an acute apex, typically 4–8 cm long and 4–6 cm wide. The leaf margin is serrate, and the surface shows a hairy nature under a microscope (Figure 2A,B). The leaf surface shows scarce strigose-type non-glandular trichomes on the upper side, and an abundance of tomentose-type trichomes on the lower surface. The leaf is hypostomatic, with numerous stomata present on the lower (abaxial) surface (Figure 2C,D). Stomata are anomocytic, with guard cells surrounded by epidermal cells, which are slightly differentiated from the normal epidermal cells. The guard cells appear elevated and form dome-like structures. The transverse section of the leaf is characteristic of a single-layered, thick epidermal cell composed of polygonal isodiametric cells with straight anticlinal walls. The mesophyll layer consists of two layers of parenchyma cells—one is palisade parenchyma, two cells thick, and rich in chloroplasts and calcium crystals. The lower region of the mesophyll consists of spongy parenchyma cells with intercellular spaces (Figure 2E–L). The vascular bundle in the midrib is prominent and slightly bulged on the adaxial side, capped with the presence of collenchyma cells. A few layers of sclerenchymatous cells surround the vascular bundle; it is visible at the abaxial side. In the center, the xylem is located towards the adaxial, while the phloem is found in the abaxial. The xylem cells are lignified and exhibit strong fluorescence under epifluorescence observations (Figure 2F,H). The upper and lower epidermal cells contain an abundance of calcium oxalate crystals, mostly in prismatic crystal shapes, along with some irregular shapes (Figure 2L).

#### 2.2.2. Stem Anatomy of *C. mexicana*

**Figure 3:**
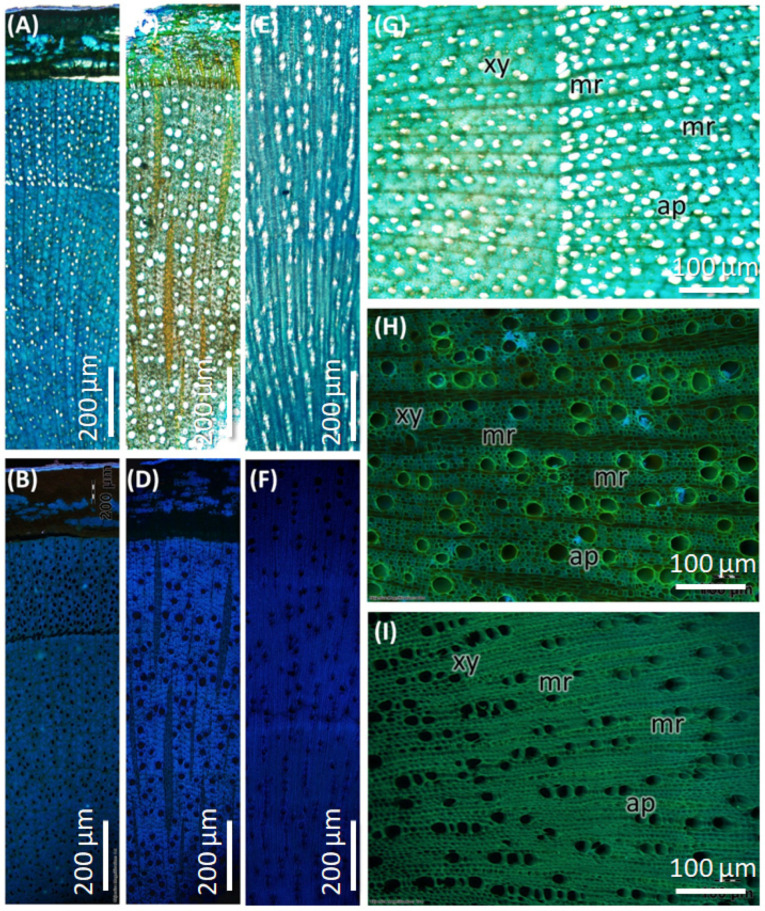
Transverse section of stem and root of *C. mexicana* compared with the market sample under bright-field microscopy, stained with TBO. (**A**,**B**,**G**) stem, (**C**,**D**,**H**) root, and (**E**,**F**,**I**) market sample (stem wood). xy: xylem, mr: medullary rays, ap: axial parenchyma. Scale bar: (**A**–**F**) 200 µm and (**G**–**I**) 100 µm.

**Figure 4:**
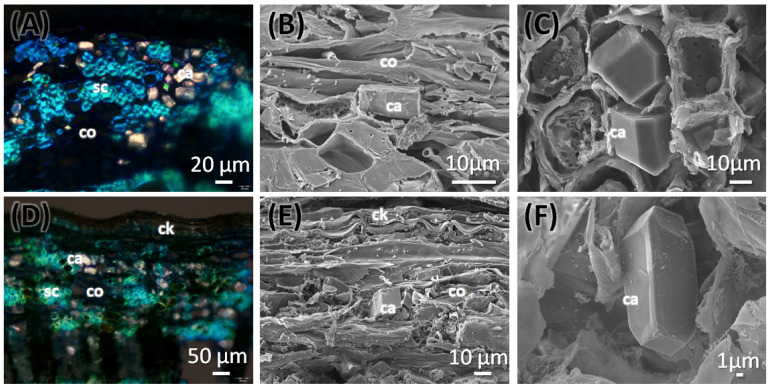
Transverse section of stem (**A**–**C**) and root (**D**–**F**) of *C. mexicana* showing the outer cork and cortex regions, viewed under polarized light (**A**,**D**) and scanning electron microscope (**B**,**C**,**E**,**F**). ca: calcium, ck: cork, co: cortex, sc: sclereids. Scale bars: (**A**) 20 µm, (**D**) 50 µm, (**B**,**C**,**E**) 10 µm, and (**F**) 1 µm.

**Figure 5:**
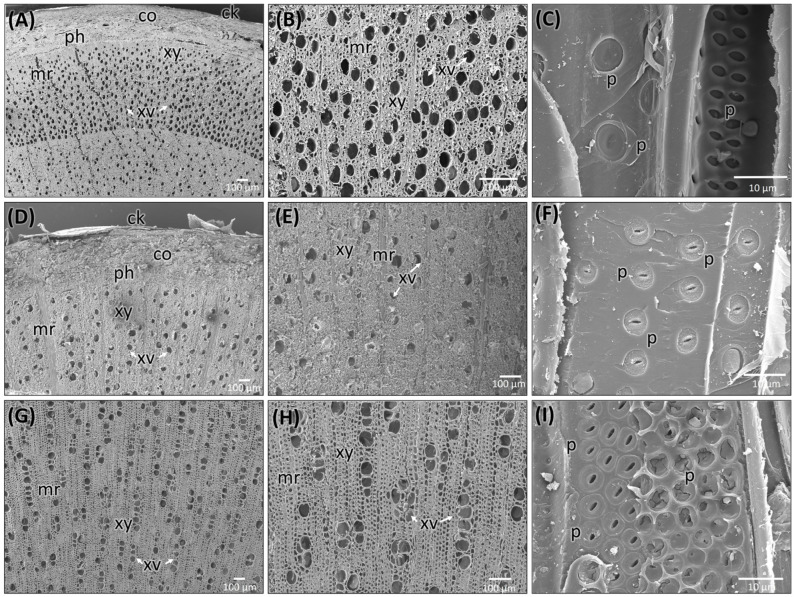
Scanning electron microscopic images of stem (**A**–**C**) and root (**D**–**F**) of *C. mexicana* and its adulterated market sample (**G**–**I**). ck: cork, co: cortex, ph: phloem, xy: xylem, mr: medullary rays, xv: xylem vessels, p: pits. Scale bars: (**A**,**B**,**D**,**E**,**G**,**H**) 100 µm and (**C**,**F**,**I**) 10 µm.

**Figure 6:**
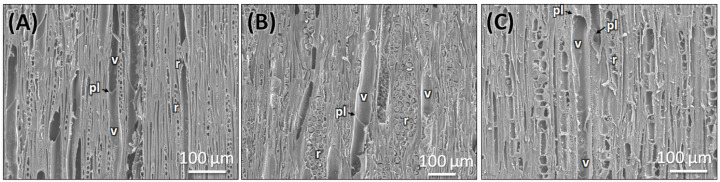
SEM observation of TLS section of genuine stem (**A**), root (**B**), and commercial sample (**C**). v: vessel, pl: perforation plate, r: ray cells. Scale bars: (**A**–**C**) 100 µm.

The stem of *C. mexicana* displays well-defined secondary growth characteristics typical of dicotyledonous plants. The transverse section of the stem consists of the cork layer, which is prominently developed, consisting of 4–6 layers of compactly arranged cells with a width of 50 ± 15 µm. A multi-layered cortex (10–15 cells thick of 150 ± 20 µm) follows, containing grouped sclereids patches that provide mechanical support (Figure 3A,B and Figure 4A) and shows the presence of calcium crystals (Figure 4A–C). Both cork cambium and vascular cambium are present and active, each 2–3 cells thick. The wood is semi-ring porous, with distinct growth ring boundaries. Vessels differ in size and density between earlywood and latewood, which is clearly visible in each annual ring, especially near the boundaries. The vessel diameter ranges from 10 µm (minimum) at the end of one annual ring to a maximum of 55 µm at the beginning of another annual ring, and they are arranged in organized patterns. Vessel pits are bordered and arranged alternately. Medullary rays are 2–3 cells thick (Figure 3A,B,G, Figure 5A–C and Figure 6A). Prismatic calcium oxalate crystals are notable in the region of cortex and wood medullary rays. Pith is present in the stem, providing a key distinguishing feature from the root.

#### 2.2.3. Root Anatomy of *C. mexicana*

The transverse section of the root shows that the cork consists of 2–3 cell layers with total width of 80 ± 15 µm, and the cortex is 10–15 cells thick, measuring 200 ± 20 µm in width. Lignified grouped sclereid patches are present in the cortex region (Figure 3C,D and Figure 4D). The presence of prismatic calcium crystals is evident in the cortex. The cork cambium and the vascular cambium were composed of 2–3 cell layers, respectively. The wood is diffuse-porous, with vessels that are solitary and arranged unevenly. There is a minimal distinction between earlywood and latewood vessels. Vessel pits are bordered and scattered (Figure 3C,D,H). The axial parenchyma cells are diffused and apotracheal. The medullary rays alternate between wider bands, 5–6 cells across, and two cells thick. (Figure 3H and Figure 6B), with the scanning electron microscopy giving the more detailed structural observations (Figure 5D–F and Figure 6B).

#### 2.2.4. Anatomy of the Market Sample

The dried market sample sold under the name *Raíz de Tejocote*, presumed to be root of *C. mexicana*, lacks several key diagnostic features observed in authentic root and stem tissues. The transverse section of the sample exhibits an absence of cork and cortex, as well as the sclerenchyma patches and cambial tissues, suggesting the sample is highly processed by peeling off the bark portion. The wood is diffuse-porous, like the root of genuine *C. mexicana*. Still, vessels are solitary, diffused, and radially arranged in rows of two to three adjacent vessels (Figure 5G,H and Figure 6C), differing from the random distribution seen in the root and the semi-ring-porous pattern in the stem. The vessels are clustered and bordered, and medullary rays are significantly reduced to only a single cell in thickness. These characteristics differ from those of the true stem of *C. mexicana*. These observations indicate that the material marketed as Raíz de Tejocote does not correspond to either the root or stem of *C. mexicana* (Figure 3E,F,I and Figure 5G–I).

Based on macro- and microscopic characteristics, the key comparative features between the stems and roots of *C. mexicana* and the market sample were highlighted.in the Table 1.

#### 2.2.5. Energy-Dispersive X-Ray Spectroscopy (EDS) Analysis

**Figure 7:**
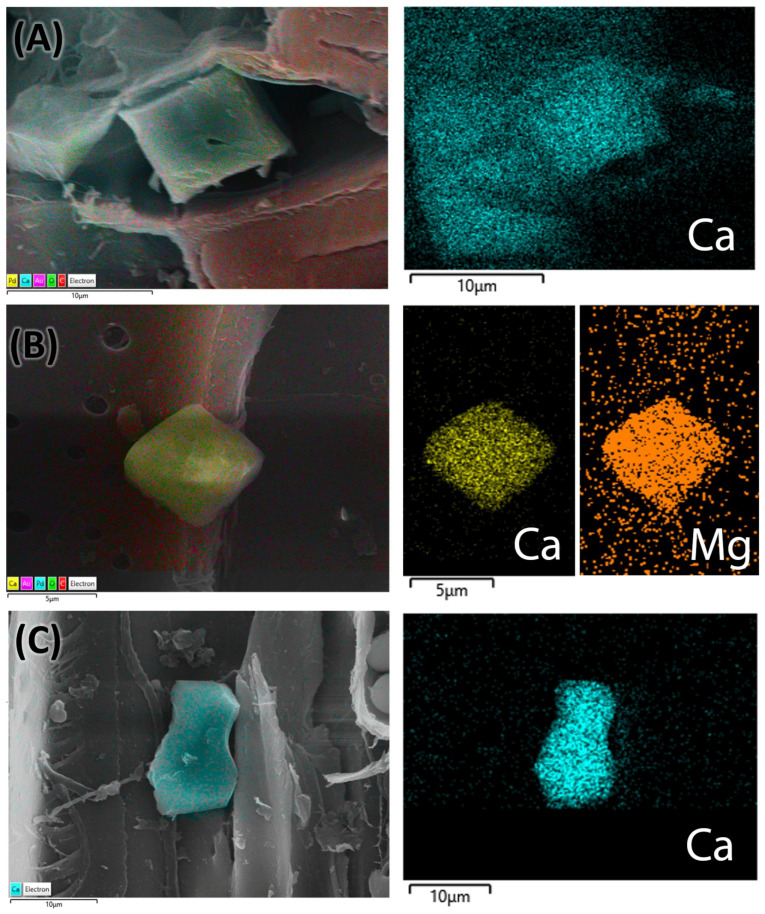
EDS analysis mapping images of the root (**A**) of *C. mexicana*, and the adulterated market sample (**B**,**C**). The pseudocolor image shows the calcium crystals and their different elemental compositions with various colors, visualized using AZtecLive software (Version 6.1). Scale bar: (**A**,**C**) 10 µm and (**B**) 5 µm.

The presence of calcium crystals was analyzed to determine their elemental composition using energy-dispersive spectroscopy (EDS) techniques in conjunction with scanning electron microscopy (SEM). The structural shape of the genuine *C. mexicana* is prismatic (Figure 7A), while variations in the calcium crystals from the market samples were noted, suggesting that these crystals may take on a bipyramidal form (Figure 7B) or exhibit prismatic variance (Figure 7C). EDS spectra revealed prominent peaks corresponding to calcium (Ca Kα at approximately 3.69 keV), confirming its significant presence within the crystalline structures. The structural and elemental mapping composition is presented in Figure 7. These crystals were predominantly located within the wood’s parenchymatous tissues and in the bark and cortex regions of the genuine root section of *C. mexicana*. They were observed in high abundance across both genuine and market samples.

## 3. Discussion

The presence of *Crataegus* species around the world is over 200 species; only 13 species are distributed in the region of Mexico, namely, *C. aurescens* J.B.Phipps, *C. baroussana* Eggl., *C. cuprina* J. B. Phipps, *C. gracilior* J. B. Phipps, *C. grandifolia* J. B. Phipps, *C. greggiana* Eggl., *C. johnstonii* J. B. Phipps, *C. mexicana*, *C. nelsonii*, *C. rosei* Eggl., *C. stipulosa* (Kunth) Steud. (syn. of *C. mexicana*), *C. sulfurea* J.B.Phipps, *C. tracyi* Ashe ex Eggl., and *C. uniflora* Münchh. One species, namely *C. pubescens*, is a synonym of *C. gracilior*, and the species *C. serratissima* J. B. Phipps, is unplaced according to the current database of Kew’s Plants of the World online due to the name is not validly published to establish a clear identity [19].

The leaf exhibits a distinct stomata arrangement, with dome-like projected guard cells surrounded by undifferentiated epidermal cells; these characteristics have been observed previously as well [20]. The vascular bundle in the leaf is collateral, a characteristic also shared by the species *C. ambigua* [21]. However, since *C. ambigua* is native to Ukraine to East Türkiye and Caucasus and not native to Mexico, it is unlikely to be confused with *C. mexicana*.

The differences in external morphology among these samples are apparent, especially in terms of color and texture. The market sample appears pale, white, and lightweight; the chopped pieces lack the bark portion. These features suggest that the sample most likely comes from the stem, since the genuine root is red-brown. The observed morphological traits, such as the presence of a pith, indicate that the material originates from the central stem area, but is not the same as a genuine *C. mexicana* stem based on the color, texture, and wood hardness. 

The anatomical comparison between the roots and stems of *C. mexicana* and market sample highlights several key differences crucial for authentication. Notably, the cork, cortex, sclerenchyma, and vascular tissues (cork cambium and vascular cambium) are present since these samples are genuine and present as a whole sample in both the root and stem, but are completely absent in the market sample. It is also evident that the market sample consists of only the wood region of the stem, not including the bark. The market sample is pale white and contains a pith region, indicating that it is derived from the stem, not the root. The anatomical characteristics of the wood further confirm that the sample does not represent differentiated heartwood and sapwood, as evidenced by the presence of pith. Further, the wood type differs: the stem shows a semi-ring-porous structure with distinct growth ring boundaries, while both the root and market sample exhibit a diffuse-porous wood, but the porous arrangements are different between the root of *C. mexicana* and the stem structure of the market sample, with indistinct or absent growth rings and size and density of the vessels. Considering the vessel characteristics, the arrangement in the stem is organized by size into earlywood and latewood. In contrast, in the root, vessels are randomly arranged, and in the market sample, they appear solitary, diffused, and radially aligned. Vessel pits also differ, being scattered in the root, alternate in the stem, and clustered in the market samples. Additionally, medullary rays are thick and multi-layered in the root, narrower in the stem, and reduced to a single-cell thickness in the market sample. These differences strongly indicate that the market sample lacks the structural integrity of authentic *C. mexicana* stem or root tissues. Comparisons of wood characteristics with those of other *Crataegus* species from Mexico are limited in the literature. In contrast, some studies on wood anatomy in other species from different countries were noted for comparison. For example, *C. azarolus* exhibits diffuse-porous wood with occasional multiple-vessel groupings in the radial direction, homogeneous rays, vessels with simple perforation plates, and alternate inter-vessel pits [22], features also observed in *C. tanacetifolia* (Lam.) Pers. and [23] *C. mexicana*. Thus, wood characteristics such as diffused porous, alternate vessel pits, simple perforation plates, 2–3 rays, and the presence of prismatic crystals are very common in the genus *Crataegus*. The wood characteristics of *C. monogyna* exhibit variations in vessel sizes, as well as sub-characteristics of the wood’s axial parenchyma, such as diffuse aggregates. When compared with the characteristics of the wood, such as the growth rings, which are distinct in *C. mexicana* and *C. monogyna,* but indistinct in *C. laevigata*, all three species show diffused-porous wood [24,25], but in our sample, designated as semi-ring porous, the difference in vessel size is close to the annual ring boundaries.

For comparison, the key anatomical characteristics of the wood of the studied species were evaluated against those of related taxa reported in the literature, and the summarized results are presented in Table 2 below.

Comparative analysis indicates that *C. mexicana* is readily differentiated by its semi-ring-porous stem wood and the presence of diffuse axial parenchyma, traits that contrast it from species such as *C. douglasii*. Furthermore, *C. mexicana* exhibits a markedly higher accumulation of gums and phenolic substances relative to the other species examined.

There has been limited classification of the anatomical characteristics of the stem and root of *C. mexicana* before this study, and there is no specific literature on the botanical adulteration of this species. However, some descriptions of wood anatomy have provided valuable comparisons for the anatomical features analyzed in the present study. There is a record on its sapwood, which is cream in color, and the heartwood is dark brown to reddish brown. This is similar to the heartwood from the study samples as well. The anatomical study of the stem shows vessels that are exclusively small and numerous [28]. This characteristic was also noted in our study. The axial parenchyma of the stem is noted as diffuse in aggregates [25], which is different in the market samples. The EDS analysis shows the elemental composition of the crystals present in both the genuine and market samples, indicating that calcium and magnesium are part of the crystal structure.

The overall summary of this study highlights the importance of microscopic characterization in authenticating botanical raw drugs. The evaluation of *C. mexicana* stem and root is distinctly different from the adulterated stem portions of various species sold in the market as Tejocote. Samples sold as Tejocote or Raíz de Tejacote in Sonora market, Mexico, consist of stem pieces labeled as roots. This sample cross-verification is representative of the adulteration issue. This manuscript highlights the presence of stem pieces sold as roots in the market.

## 4. Materials and Methods

### 4.1. Botanical Materials

All fresh, authentic stems, roots, and leaves of *C. mexicana* were collected at San Antonio Zoyatzingo, Estado de México (19°11.484′, 098°48.777; 2504 m above sea level), and recorded using a Garmin eTrex^®^ 20 GPS (Garmin, New Taipei City, Taiwan, China). The identification and authentication of the plant were carried out by M. en C. Santiago Xolalpa, a botanist of the Herbarium of Medicinal Plants of the Instituto Mexicano del Seguro Social and deposited with the number IMSSM 17222. A sample labeled as Tejocote root was purchased at the Sonora Market, Mexico City, in November 2024 and included in this study. The plant materials were assigned unique NCNPR numbers—for *C. mexicana*: NCNPR #: 25833 and for the market sample NCNPR #: 25832—and deposited at the Botanical Repository of the National Center for Natural Products Research, University of Mississippi, USA.

### 4.2. Preparation of Samples for Macro- and Microscopic Studies

The collected samples were subjected to shade drying and followed by hot air oven drying. The dried samples were fixed with FAA (Formaldehyde–Alcohol–Acetic Acid—10%:50%:5% + 35% water) before being processed for detailed anatomical studies, and several sections of 20 to 25 µm were hand-cut using a razor blade, and were stained with an aqueous solution toluidine blue O (TBO), containing 0.05% in 0.1 M phosphate buffer at pH 6.8 for basic histology and auramine O 0.5% dissolved in 70% ethanol for fluorescence observation [29,30,31]. All mounts were prepared on glass slides with water. Photomicrographs were obtained using an Olympus BX53 compound microscope (Olympus Corp., Tokyo, Japan) equipped with an Olympus DP74 camera system. The fluorescence observed through the UV filters (wide longpass cubes) with an excitation wavelength of 340–390 nm and a barrier filter of 420 nm was used for fluorescence imaging. Images were processed using CellSens standard imaging software version 3.1, build 21199.

### 4.3. Preparation of Samples for Scanning Electron Microscopy (SEM) Observation

The specimens were fixed in FAA (Formalin/Glacial Acetic Acid/Ethanol/DI water) at the following percentages: 10% Formalin/5% Glacial Acetic Acid/50% ethanol (using absolute EtOH)/35% deionized (DI) water [32]. Then, the sample was washed with running water and passed through a series of ethanol solutions with concentrations of 30%, 50%, 70%, 90%, and 100% for 30 min per step. The processed samples were dried using a Leica CPD300 critical point dryer (Leica Microsystems, Wetzlar, Germany) supplied with liquid CO_2_. Dried samples were mounted on aluminum stubs with double-sided adhesive carbon tape and then coated with 10 nm gold–palladium (60:40) using a Desk V HP sputter coater (Denton Vacuum, Moorestown, NJ, USA) supplied with argon gas. The samples were imaged using a JSM-7200FLV field-emission SEM (JEOL Ltd., Tokyo, Japan). The sample imaging conditions included 10 keV, a working distance of 8 to 10 mm, and the lower electron detector in SE mode. The elemental composition of calcium oxalate crystals was detected with an energy-dispersive X-ray spectroscopy (EDS) detector (Oxford Instruments, Oxford, UK) mounted on the JSM-7200FLV instrument. The EDS detector was operated at around 25 keV, with the scan mode set to mapping [33], and analyzed using AZtecLive (Version 6.1, Oxford Instruments NanoAnalysis) software.

## 5. Conclusions

This study provides a comprehensive anatomical comparison between genuine stem and root tissues of *C. mexicana* and commercial market samples sold as Raíz de Tejacote, with a focus on developing reliable authentication parameters. Microscopic analysis revealed the absence of key structural features: cork, cortex, sclereids, and cambial tissues in the market samples due to the absence of a bark portion, and its color indicates it is a wood portion of the stem. Additionally, variations in wood type, vessel arrangement, medullary ray thickness, and the presence or absence of pith further distinguish the genuine material from its commercial counterpart. These anatomical features can be used to identify the botanical materials sold commercially as Raíz de Tejacote. The applicability of these anatomical characteristics is limited to whole, chopped, or coarse powdered materials (mesh 80; ~150 µm). For finely powdered samples or extracts, alternative methods, including chromatographic chemical profiling or DNA analysis, are recommended for reliable identification. Further studies are required to differentiate the 13 *Crataegus* species distributed across Mexico and the United States through detailed anatomical comparison. Such investigations are crucial for the accurate authentication of *C. mexicana* (Tejocote), thereby preventing adulteration, supporting the quality control of raw materials, and ensuring compliance with regulatory requirements for the safety and efficacy of herbal products.

## Figures and Tables

**Table 1:** List of anatomical characteristics for differentiation between the stems and roots of *C. mexicana* and the market sample.

Features	Stem (Figure 5A–C)	Root (Figure 5D–F)	Market Sample (Figure 5G–I)
Cork	Present, 4–6 layers thick 50 ± 15 µm (mean + SD)	Present, 2–3 layers thick 80 ± 15 µm (mean + SD)	Not found
Cortex	10–15 cells thick,150 ± 20 µm (mean + SD)	10–15 cells thick, 200 ± 20 µm (mean + SD)	Not found
Sclerenchyma in Cortex	Present, in grouped patches	Present, in grouped patches	Not found
Cork Cambium (CC)	2–3 cells thick	2–3 cells thick	Not found
Vascular Cambium (VC)	2–3 cells thick	2–3 cells thick	Not found
Wood Type	Semi-ring porous (Figure 5A)	Diffuse porous	Diffuse porous
Growth Ring Boundaries	Present	Indistinct or absent (Figure 5D)	Indistinct or absent (Figure 5G)
Earlywood vs. Latewood Vessels	Difference in vessel density and size	Not much difference	The difference is only in size
Vessel Arrangement	Semi-ring porous; vessels in the earlywood are distinctly	Diffuse, solitary, close-by, uneven diameter	Solitary, diffuse, Radial pattern with multiples of 2- and 3- adjacent vessels
	larger than those in the latewood of the previous growth ring		
Vessel size	30 ± 20 µm (mean + SD) in earlywood and 20 ± 10 µm (mean + SD) in latewood	50 ± 10 µm (mean + SD)	55 ± 20 µm (mean + SD)
Vessel Pits	Bordered pits, alternate arrangement (Figure 5C)	Bordered pits, scattered (Figure 5F)	Bordered pits, clustered arrangement (Figure 5I)
Axial Parenchyma	Diffuse, aggregated apotracheal	Diffuse, apotracheal	Extremely rare or absent
Medullary Rays	1–3 cells thick	Alternating 5–6 cells and two thick cells	Uniseriate
Pith	Present	Absent	Present

**Table 2:** Comparison of anatomical wood characteristics with a few other species of *Crataegus* vs. *C. mexicana* [26,27].

Wood Characteristics	*Crataegus mexicana*	*Crataegus azarolus* L.	*Crataegus douglasii* Lindl	*Crataegus germanica* (L.) Kuntze	*Crataegus hupehensis* Sarg.	*Crataegus laevigata* (Poir.) DC.	*Crataegus* *monogyna*	*Crataegus tanacetifolia (Poir.) Pers.*
Growth Ring	Distinct	Boundaries are indistinct or absent.	Boundaries are indistinct or absent.	Boundaries are indistinct or absent.	Boundaries are indistinct or absent.	Boundaries are indistinct or absent.	Distinct	Boundaries are indistinct or absent.
Wood porosity	Semi-ring porous in the stem, diffuse porous in the root	Diffuse porous	Diffuse porous	Semi-ring porous	Diffuse porous	Diffuse porous	Diffuse porous	Diffuse porous
Vessel arrangement	Exclusively solitary	Exclusively solitary	May varies	Exclusively solitary	May varies	May varies	May varies	Exclusively solitary
Perforation plates	Simple	Simple	Scalariform	Simple	Scalariform	Simple	Simple	Simple
vessel pit	Inter-vessel pits alternate	Inter-vessel pits alternate	Inter-vessel pits alternate	Inter-vessel pits alternate	Inter-vessel pits alternate	Inter-vessel pits alternate	Inter-vessel pits alternate	Inter-vessel pits alternate
pit size range	Minute to 4 µm	Minute to 4 µm	4–7 µm	4–7 µm	Medium: 7–10 µm	Medium: 7–10 µm	Minute to 4 µm	4–7 µm
Gums and deposits	Present	Absent	Absent	Absent	Absent	Present	Absent	Absent
Tracheid and fibers	Simple libriform fiber	Simple libriform fiber	Simple libriform fiber	Simple libriform fiber	Simple libriform fiber	Simple libriform fiber	Simple libriform fiber	Simple libriform fiber
Axial parenchyma	Diffuse	Diffuse	Diffuse, in aggregates	Diffuse	Diffuse	Diffuse	Diffuse	Diffuse
Rays	1–3 cells	1–3 cells	1–3 cells	1–3 cells	1–3 cells	4–10 cells	1–3 cells	1–3 cells
Mineral inclusions	Prismatic crystals	Prismatic crystals	Prismatic crystals	Prismatic crystals	Prismatic crystals	Prismatic crystals	Prismatic crystals	Prismatic crystals

## Data Availability

The data supporting the reported results are available at reasonable requests to the corresponding author.
